# Factors influencing termination of resuscitation in children: a qualitative analysis

**DOI:** 10.1186/s12245-020-0263-6

**Published:** 2020-03-14

**Authors:** Rashida T. Campwala, Anita R. Schmidt, Todd P. Chang, Alan L. Nager

**Affiliations:** 1 Division of Emergency and Transport Medicine, Children’s Hospital Los Angeles, 4650 Sunset Blvd., Mail Stop 113, Los Angeles, CA 90027 USA; 2 Department of Pediatrics, Keck School of Medicine, University of Southern California, Los Angeles, CA USA

**Keywords:** Pediatric resuscitation, Termination of resuscitation, Termination of pediatric resuscitation

## Abstract

**Background:**

Pediatric Advanced Life Support provides guidelines for resuscitating children in cardiopulmonary arrest. However, the role physicians’ attitudes and beliefs play in decision-making when terminating resuscitation has not been fully investigated. This study aims to identify and explore the vital “non-medical” considerations surrounding the decision to terminate efforts by U.S.-based Pediatric Emergency Medicine (PEM) physicians.

**Methods:**

A phenomenological qualitative study was conducted using PEM physician experiences in terminating resuscitation within a large freestanding children’s hospital. Semi-structured interviews were conducted with 17 physicians, sampled purposively for their relevant content experience, and continued until the point of content saturation. Resulting data were coded using conventional content analysis by 2 coders; intercoder reliability was calculated as κ of 0.91. Coding disagreements were resolved through consultation with other authors.

**Results:**

Coding yielded 5 broad categories of “non-medical” factors that influenced physicians’ decision to terminate resuscitation: legal and financial, parent-related, patient-related, physician-related, and resuscitation. When relevant, each factor was assigned a directionality tag indicating whether the factor influenced physicians to terminate a resuscitation, prolong a resuscitation, or not consider resuscitation. Seventy-eight unique factors were identified, 49 of which were defined by the research team as notable due to the frequency of their mention or novelty of concept.

**Conclusion:**

Physicians consider numerous “non-medical” factors when terminating pediatric resuscitative efforts. Factors are tied largely to individual beliefs, attitudes, and values, and likely contribute to variability in practice. An increased understanding of the uncertainty that exists around termination of resuscitation may help physicians in objective clinical decision-making in similar situations.

## Background

The decision to terminate resuscitative efforts in pediatric cardiopulmonary arrest is complex [1, 2] and may vary considerably among physicians and across institutions. Moreover, while guidelines for resuscitation like Pediatric Advanced Life Support (PALS) and Advanced Cardiac Life Support (ACLS) have been taught and followed by clinicians worldwide, the often subjective “non-medical” factors that could influence or guide a clinician’s decision to prolong or terminate cardiopulmonary resuscitation (CPR) are not considered. The International Liaison Committee on Resuscitation (ILCOR) has no position on non-medical factors.

Medical considerations for termination, such as initial cardiac rhythm, number of doses of epinephrine (adrenaline), and pupil response have been widely published [3–7], while more subjective “non-medical” factors, including physicians’ attitudes and beliefs, have been discussed in very few publications. Larkin reviewed the complexity of the multifactorial decision to discontinue resuscitative efforts, including futility judgments and provider experience and comfort, especially when terminating efforts in children [1]. Scribano et al. discussed medical factors influencing termination of resuscitative efforts in children and mentioned additional considerations such as child abuse, organ donation, and co-morbid conditions [2]. Meanwhile, the European Resuscitation Council (ERC) acknowledges the responsibility of the physician to weigh and balance the risks, benefits, and costs resuscitative interventions have on the patient and their family, as well as the costs to the health care system and society as a whole [8].

Variability surrounding the termination of resuscitation in children is highlighted in the Emergency Department (ED), where the duration of resuscitative efforts on pediatric patients in cardiac arrest involves significant uncertainty with vague and unclear end-points. This uncertainty may arise from such entities as having insufficient details surrounding the cardiopulmonary arrest, an unknown downtime, an unknown cause of the arrest, parental absence, or even parental interference [9].

While some of the “non-medical” factors have been described previously in the literature, to our knowledge, there has been no effort undertaken to investigate the totality of “non-medical” factors and beliefs that physicians take into account, or are influenced by, when making resuscitative decisions in children. Thus, a clearer understanding of the uncertainty that exists around termination of resuscitation may help physicians to make more objective decisions in that same context. Among other mitigation effects, this may also serve to ameliorate some of the stress and anxiety surrounding critical events and high-stakes decisions. The aim of this study, therefore, was to identify and explore the phenomena of “non-medical” considerations surrounding the decision to terminate resuscitative efforts by Pediatric Emergency Medicine (PEM) physicians.

## Methods

### Setting

This was a phenomenological study meaning that it sought to gather the perspectives of a group of people who all have experienced a shared phenomenon (event) in order to gain a better understanding of that specific occurrence [10]. The study was conducted at a single, free-standing, urban, and tertiary care children’s hospital (not physical connected with a university or other health care setting); this hospital has a level 1 trauma center designation and an ED volume of approximately 90,000 pediatric patients per year. The hospital is located in the USA. The ED averages 400 critical patients per year requiring intravenous (IV) fluids, airway management, and other resuscitative measures, 10 of which are out-of-hospital cardiopulmonary arrests. These numbers are averages for the past 5 years, during which no outlier incidents such as local mass casualties or major natural disasters had occurred. The ED is staffed by pediatric physicians board-certified or board-eligible in PEM; these physicians have completed 4 years of medical school, 3 years of general pediatrics residency, and a 3 years of PEM fellowship before attaining the position of attending physician. Our research team consisted of 2 PEM attending physicians, 1 PEM fellow physician, who was the Principal Investigator (PI), 1 non-clinical Research Manager experienced in qualitative methods, and 1 non-clinical Research Coordinator. All research activities were approved by the hospital’s Institutional Review Board.

### Selection of participants

Purposeful sampling was used to generate an information-rich group with content expertise in the subject of interest [11]. Because the study sought to paint a nuanced picture of the factors that motivate experienced physicians to terminate or prolong resuscitation, all PEM attending physicians at the institution were asked to participate in the study. The decision to exclude PEM fellow physicians was made due to their relative clinical inexperience, which is known to influence decision-making during resuscitation [12]. Of the 20 attending physicians at the study institution, 2 did not participate due to their involvement in the study and 1 was not approached as data saturation was reached. All other PEM physicians voluntarily agreed to participate.

### Interview guide

The interview guide (Appendix 1) incorporated a combination of concepts drawn from literature [2, 3, 7, 8, 12–18] and a modified Delphi method [19]. Criteria for termination were not explored in the 2009 review article by Topjian et al. [20] or in the joint technical report by the American Academy of Pediatrics and the American College of Emergency Physicians [21]. However, factors that may be considered when terminating resuscitation found in the literature included age, prognosis, futility, quality of life, parental presence, child abuse, cost to the health care system, fear of litigation, and organ donation [2, 3, 5, 12–18, 22–24].

Participants in the modified Delphi panel included a PEM attending physician, the PI, and the Research Coordinator. The group conducted a total of 4 rounds of revisions to the guide. After each subject interview, the guide was iteratively modified to generate more detailed responses from participants.

### Data collection

Subjects provided data about their level of experience and the frequency of leading and terminating pediatric resuscitations. The PI and/or the Research Coordinator interviewed each participant. While in some circumstances, the familiarity of interviewer and subject may be considered a source of bias, and in many qualitative studies, this familiarity is considered an advantage [25]. In this case, the PI’s experience allowed for greater insight and validity checking with subjects throughout the interviews. All interviews were recorded and subsequently transcribed by the PI and 2 research assistants. All of the transcripts transcribed by the research assistants were then reviewed by the PI to account for accuracy.

### Data analysis

Transcripts were entered into NVivo, a qualitative data management software (QSR; Burlington, MA, version 11 Pro, 2015), and the data analysis team consisted of the PI and the Research Manager. The decision to have multiple coders was made in order to establish content validity [26]. Because of the PI’s familiarity with the subject matter and clinical working relationship with the subjects, the position of second coder was purposefully assigned to a non-clinician. The analytical approach selected for this study was conventional content analysis; this approach involves line-by-line inductive coding, completed over multiple rounds. This was chosen given the lack of existing literature on the topic [27, 28].

Consistent with the procedures of conventional content analysis, the coding team independently open-coded the first 3 transcripts. To do this, each coder inductively created and assigned codes, line-by-line through the transcripts, generating 2 extensive lists of codes. The team then met over several sessions and iteratively refined the codebook, reaching consensus for the coding of the first batch of transcripts and assigning a definition to each code. General themes and initial code relationship structures were established. To test coding reliability [26], the team used the completed codebook to independently analyze 2 additional transcripts and then came together to compare coding structures. Intercoder reliability was calculated through the NVivo software (k = .91). To resolve coding disagreements, 2 authors were consulted and a discussion took place until consensus was reached. The completed codebook is available in Table 3 in Appendix 2.

The remaining transcripts were divided among the 2 coders and independently coded. The projects were then merged in NVivo, and the team members divided the list of codes in order to perform code cleaning. This is the process of looking at each of the instances in which an individual code is used and ensuring that the code is applied consistently throughout the dataset [26]. The team then met and refined the coding structure; codes were grouped within parent codes based on a common theme, or collapsed into each other so that each code represented an individual idea. As theme development progressed, some codes were re-named and final edits to the relationships of the codes were made.

To conduct member checking [29], transcripts were returned to 20% of participants to verify accuracy of transcription and to confirm that data analyst interpretations were accurate.

The consolidated criteria for reporting qualitative research (COREQ) [29] was adhered to during study design, data collection, and analysis, as outlined in Table 4 in Appendix 3.

## Results

### Demographics

Seventeen attending physicians in the Pediatric Emergency Department (PED) were interviewed. The subject characteristics are summarized in Table 1. Sixteen (94%) participants were full-time PEM attending physicians who had training in both general pediatrics and PEM, while one participant was a part-time PEM physician who also had training in both general pediatrics and PEM. Despite the average number of documented cardiopulmonary resuscitations in the ED (average of 10 per year), all but 4 participants (76%) self-reported that they led greater than 3 pediatric cardiopulmonary resuscitations on average per year. Only 2 (12%) physicians recalled that they did not experience an arrest that required pronouncement of a patient (i.e., termination) in the past year and also had 3 or fewer terminations in the last 5 years. Thirteen (76%) subjects were parents of children ranging in age from 18 months to 19 years. Forty-one percent of physicians interviewed were male, and 59% were female.

**Table 1:**
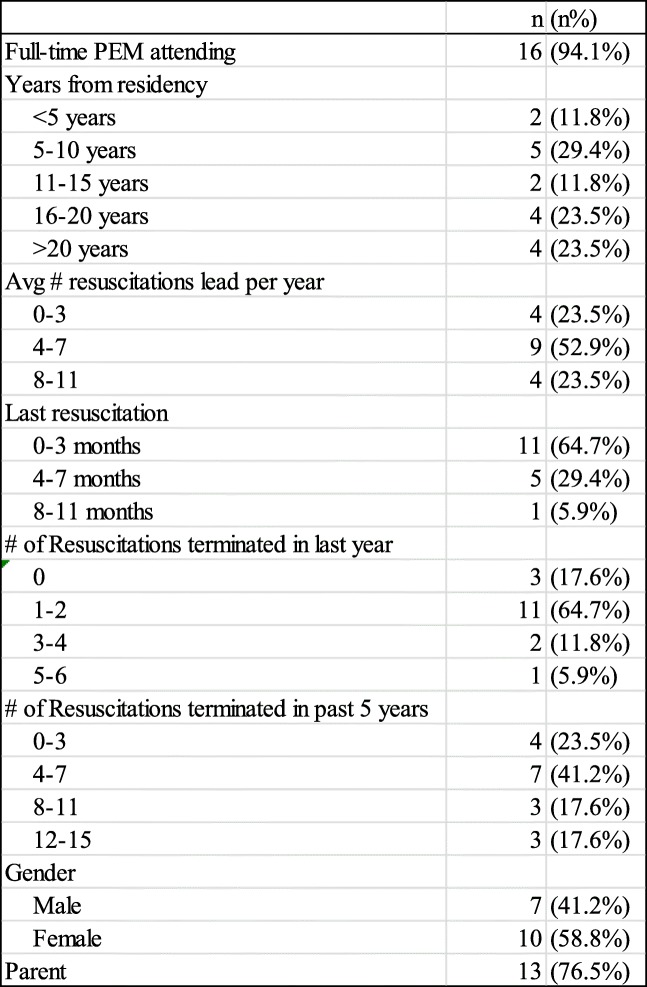
Demographics of the participants

### Coding structure

The coding process yielded 5 broad categories of factors that influenced physicians’ decision to terminate resuscitation. These were *legal and financial factors*, *resuscitation-related factors*, *parent-related factors*, *patient-related factors*, and *physician-related factors*. There were a total of 78 unique factors across all 5 categories (Table 2). Additionally, 4 directionality codes were developed in order to better answer the study question and also identify which factors influenced physicians to terminate a resuscitation, prolong a resuscitation prior to termination, or which factors were not considerations in the decision-making process (Table 5 in Appendix 4). Each of the 78 factors were double-coded with a directionality code when applicable. The directionality codes identified were *induce to prolong*, *induce to terminate*, *non-factor*, and *will not initiate CPR*. An exploratory code, *induced an emotional response*, was applied when a physician made reference to having an emotional reaction that did not otherwise influence their resuscitation. The full coding structure, including all themes and factors, can be found in Table 3 in Appendix 2.

**Table 2:**
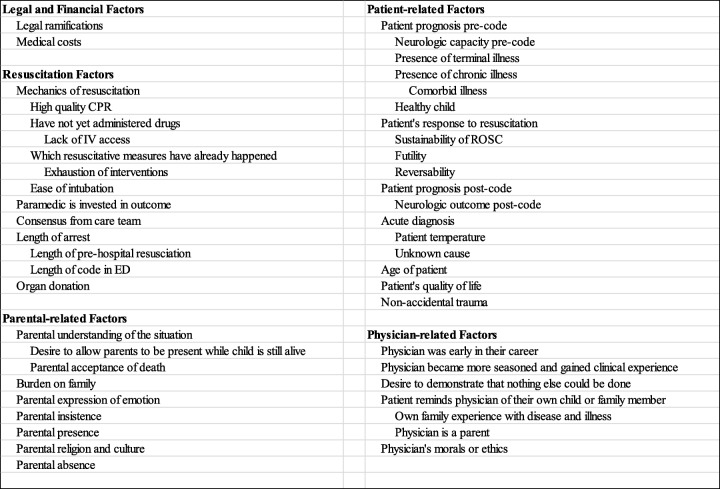
Factors most commonly stated and those affecting prolongation or termination of a resuscitation

#### Legal and financial factors

When asked about *medical costs and legal ramifications*, physicians unanimously stated that both are non-factors and that these considerations would not alter their medical decision-making process during a resuscitation.

Box 1

**Table:** 

“I don’t think about [medical costs] in that moment [of an emergent situation], but I certainly think about it all the time otherwise.” “I try not to let [legal ramifications] affect my decision-making. As long as I’m doing the right thing, even if I get sued, I’ll be protected.”	

#### Resuscitation-related factors

All physicians discussed the influence of *length of arrest* in their decision to terminate resuscitation, noting that a longer down time would make them more likely to terminate. Explanations for this included the unlikelihood of a *sustainable return of spontaneous circulation* and the likelihood of poor *neurologic outcome*. When discussing patient downtime, approximately half of the physicians discussed the *length of the arrest in the ED* while the other half spoke broadly about the total *length of arrest*, including down time prior to the arrival of Emergency Medical Services (EMS). Only once was *length of pre-hospital resuscitation* mentioned as a distinguishing factor.

While *length of arrest* was an important factor for all physicians, there was no consensus as to how long a resuscitation should continue in the ED prior to termination. Several physicians stated that they would typically stop after 20 min of resuscitation because, beyond that point, efforts are likely *futile*. Most participants, however, would not provide an estimated average length for their resuscitations and instead said that the *mechanics of the resuscitation* are more important to them. This included whether or not *IV access was obtained*, *medications were given*, *intubation was performed successfully and quickly*, and whether *all other interventions were exhausted*. If front-end efforts were strong, if there was *high-quality CPR*, and if the overall *mechanics of the resuscitation* went well, subjects were more likely to terminate quickly. Conversely, if any of these factors were perceived to be less than ideal to the treating physician, they would be inclined to prolong the resuscitation.

Another factor mentioned by approximately half of the physicians was *consensus from the care team*. If all members of the care team agreed to terminate the resuscitation, the physician would be more likely to cease resuscitative efforts. While only a few physicians stated that *organ donation* would influence them to prolong a resuscitation, the majority stated that it was a non-factor, citing that it is logistically difficult to keep a child alive purely for organ donation, especially if efforts have been futile.

One physician mentioned that they would prolong a resuscitation for the sake of the *paramedic who is invested in the outcome*. This physician gave an example of prolonging resuscitating an infant who was known to be dead on arrival to the ED medical team because the paramedics hoped for a positive outcome.

Box 2

**Table:** 

*Length of Arrest.* “… We always consider how long the patient has been down … How long the patient has been without vital signs or without … spontaneous circulation. If it’s already been a significant amount time … ultimately that patient’s outcome will not be so great even if we get the patient back. So time down is one of the biggest ones.” *Mechanics of the Resuscitation.* “… if you’ve intubated and have good access, and you have a good, cohesive staff that’s working, then you know that the front-end efforts have been strong; then I’m more likely to terminate quickly.”	

#### Parent-related factors

When asked about *parental presence*, many physicians stated that the presence of family was a non-factor and that it would not influence them to prolong or terminate the resuscitation sooner. However, several physicians felt that it was important for parents to see the effort made so that they would have an easier time *accepting the death of their child*. Furthermore, multiple physicians reported that they would prolong resuscitation due to *parental insistence* or *expression of emotion*.

While discussing *parental absence* at the bedside, the majority of physicians stated that they would prolong a resuscitation for a couple of minutes until the family could get to the ED, feeling that it was important to have the *parents present while the child was still alive* and *demonstrate to the parents that nothing else could be done*. However, a minority of physicians stated that parental absence was not a factor in their decision-making and that they would terminate resuscitation even in the absence of parents or family members. These physicians expressed that it was not “fair” to the child to prolong futile attempts at resuscitation.

Most physicians indicated that a *family’s religion and culture* would not affect their decision-making during termination of resuscitation, stating that in that moment of crisis the family’s personal beliefs would not be a consideration.

Box 3

**Table:** 

*Parental Presence.* “They need to see what we do … that we are really trying; that despite our best efforts, the outcome is not good. It’s so much harder to accept if you are later told your child died. [It is] so much harder because you didn’t see any of it, you didn’t experience any of that. So, for me, parental presence doesn’t affect my decision making, but on the flip-side, I think it’s important for the family to be there.” *Parental Absence.* “As tragic as it is, I would terminate the resuscitation. It is not in the best interest of the patient, who now has expired, to keep prolonging something, to keep going on for some poorly-defined reason.” *Parental Insistence.* “Sometimes I have prolonged a resuscitation because the parents would say, ‘You can’t end my baby’s life. He’s all that I have.’”	

#### Patient-related factors

Most participants felt that the *age of the patient* was a non-factor, and it would therefore not affect their decision to terminate resuscitative efforts.

Few physicians stated that the presence of *chronic medical problems*, such as cerebral palsy with tracheostomy and gastrostomy tube, would not affect their decision-making. However, the majority reported that they consider the general medical situation and the *acuity* of the event, and distinguish between *previously healthy children* and those with *chronic medical problems* or a *terminal illness*. These physicians felt that a healthy child is more likely to have a *reversible condition* and thereby have a better outcome than a child with multiple *comorbidities* and chronic health care needs. Therefore, they would likely prolong resuscitation in a previously healthy child and terminate relatively sooner in a patient with chronic medical problems or a terminal illness.

*Neurologic outcome post-cardiopulmonary resuscitation* and *quality of life* were factors that elicited notably strong viewpoints from a majority of physicians. A segment of the study sample felt that it was not up to them to make the judgment of what constitutes “good” quality of life. They stated that they would continue and ultimately terminate a resuscitation without much consideration regarding the patient’s quality of life post-arrest. The majority of participants, however, reported that the likelihood of a poor neurologic outcome and subsequent poor quality of life would motivate them to terminate a resuscitation. Several physicians expressed that it was precisely their duty to make this determination for the family, as parents do not often possess the same insight as physicians regarding the *burden* of having a neurologically devastated child.

*Non-accidental trauma* was unanimously reported to be a non-factor. Several physicians reported that child abuse may *induce an emotional response*, but that it would not impact their decision-making.

Box 4

**Table:** 

*Age of Patient.* “I think a 2 month old affects me the same way … a 5 year old affects me, the same way a 15 year old affects me.” *Healthy Child* versus *Child with Chronic Illness.* “A child who has been chronically ill, who has been struggling for some time with various medical problems … maybe we just can’t save this patient, versus if this was a healthy child who fell from a third story window … maybe there is some intervention we can provide quickly in order to allow this patient to have some reasonable outcome. We tend to work a little longer, a little more aggressively with trauma patients that were previously healthy, because we feel that, whether it’s right or wrong, the patient is more savable. That may not be ethically correct, but that is sort of how it’s perceived.” *Neurologic Outcome Post-Cardiopulmonary Resuscitation* and *Quality of Life.* “Everybody’s idea of quality of life is different, and it also changes over time. A family member may have initially thought that somebody who is not completely neurologically intact has a lower quality of life. After they have a child like that and have adjusted to that as their reality, they might disagree with their original decision, and now feel that their child’s quality of life, while it might not be the same as everybody else’s, is still worth it.” “We have the ability to keep people alive for years and years, but they become a burden not just to the medical system but, perhaps, to their family too. It’s so difficult because the families don’t have that perspective or insight usually.”	

#### Physician-related factors

Most physicians noted that they were more likely to prolong resuscitation *earlier in their career* and that their resuscitations have shortened in duration as they *became more seasoned and gained clinical experience*. This was generally attributed to the ability to recognize futility more rapidly and increased comfort with making difficult decisions on their own without the safety net of a more senior physician.

Another factor uncovered was that, at times, the *patient reminds the physician of their own child or family* member. One physician stated that the emotional response elicited in resuscitating a child occurred because it reminded him of his own daughter, which led him to prolong the resuscitation. Conversely, most treating physicians interviewed felt that they were able to divorce themselves from emotional attachments in order to provide appropriate medical care.

When asked if their own *morals or ethics* affected termination of resuscitative efforts, a few responded no, stating that they try to be as objective and personally removed from the situation as possible. However, the majority of physicians responded that their own morals and ethics do influence their decisions during resuscitations, stating that one’s beliefs are so deeply rooted that it is impossible to separate oneself from those views. These morals and beliefs extend to their personal definition of what is considered “good quality of life” for the patient and their family and whether this factor should be a consideration in terminating resuscitative efforts.

Box 5

**Table:** 

*Physician’s clinical experience.* “I think everybody has that trajectory. In the beginning it’s very hard to “call it” because you are the only person saying this patient is now dead. It’s a very difficult decision. Then you gain more experience and you have a little bit more insight and perspective. You can more readily get to the same conclusion as you would have gotten to when you were a fresh attending.” *Physician’s morals or ethics.* “I try not to let my own moral values interfere. Just because I have certain moral values doesn’t mean that child’s family is going to have those moral values. I’m there to serve them. I’m not there to serve my moral values.” “I come from a background where life is considered important, and so I hesitate to make decisions regarding neurologic outcomes. I’d say preservation of life is probably higher on my agenda.”	

## Discussion

Resuscitation education has, for years, focused on medical interventions taught to Emergency physicians through courses such as PALS and ACLS, but these guidelines make no mention of the “non-medical” considerations often involved in terminating resuscitative efforts. Few other decisions in medicine involve such complex and charged decisions as the irreversible decision to end life. As a result, it is essential that physicians be equipped with any information that could help them make well-conceived and thorough decisions during resuscitation. To aid in this process, we conceived the first study, to our knowledge, which conducted an in-depth exploration into the breadth of “non-medical” factors that physicians consider when making the decision to terminate resuscitative efforts in children.

In this study, physicians reported that a wide variety of considerations influence their decision to prolong or terminate resuscitation, which fell into the following categories: *legal and financial factors*, *resuscitation-related factors*, *parent-related factors*, *patient-related factors*, and *physician-related factors*. However, the relative weight and importance of each of the themes and factors varied between physicians.

A novel and interesting factor that emerged in this study was the idea that the patient reminded the physician of their own child or family. While the majority of physicians stated that this factor would only affect them emotionally and not cause them to prolong a resuscitation, it is conceivable that in a stressful and emotionally charged resuscitation, physicians may be swayed by their own emotions of sorrow for the family, distress over the loss of a child’s ability to experience life, or even fear of declaring death. This factor may be unique to resuscitating pediatric patients, as it is likely that the parental role is the nidus for these emotions and responses. Physicians’ emotions were another factor revealed in this study, described by participants as often causing them to prolong resuscitation. This underscores the significant influence non-medical factors have in medical decision-making and their role in contributing to variability in clinical practice. This variability is neither positive nor negative, but rather inevitable given each practitioner’s individual beliefs and experiences, and the uniqueness of each case.

Patient age was unanimously described in our study population as a factor not affecting decision-making during resuscitation. This may be due to the pediatric training obtained by all of the participants specializing in and actively practicing PEM, thereby increasing their comfort level. Patient age has been shown to be an important factor when making resuscitation decisions in adults, in that physicians may be less inclined to consider resuscitation in elderly patients [30]. The converse has also been shown to be true, where pediatric resuscitations are longer than those on adult patients by non-PEM physicians [31, 32]. It has also been shown that practitioners with pediatric training tend to have a shorter duration of resuscitation time than General Emergency Medicine (GEM) physicians, in that PEM physicians are twice as likely to terminate by 25 min if no return of spontaneous circulation is observed [2].

Another factor noted in the literature is whether or not the parents’ attendance at the bedside is beneficial or disruptive and, more specifically, whether it impacts resuscitation timing or efforts [9, 22, 32]. Medical societies, including the ERC, have recommended parents be at the bedside, stating that that their presence is neither disruptive nor stressful to the staff. Their proximity helps the parents to gain a more realistic understanding of the resuscitation and allows them to better grieve and adjust after their child’s death [3]. Despite these recommendations, Tripon et al. found that the majority of Emergency physicians and nurses surveyed in their study were reluctant to have parents present due to psychological trauma for the parents, risk of interference with medical management, care team stress, and, above all, a personal attitude espousing medical paternalism. Our study found that while physicians felt that parental presence was important for numerous reasons, including parental acceptance of death, most participants identified parental presence as a factor not affecting their decision to prolong or terminate a resuscitation. Moreover, the majority of physicians stated that they would prolong resuscitation in the instance of parental absence at the bedside in order to allow time for the parent to arrive to the Emergency Department.

Rules or guidelines for the non-medical considerations for terminating resuscitation in the pediatric population are sparse. The ERC suggests that the resuscitation team leader should consider terminating a resuscitation after 20 min. In addition, they outline other relevant considerations including age, cause of arrest, pre-existing medical conditions, duration of untreated cardiopulmonary arrest, and medical factors such as number of doses of epinephrine (adrenaline), the end-tidal CO_2_ value, and the presence of a shockable rhythm [3–6, 33]. In our study, there was no consensus as to how long a resuscitation should continue in the ED before termination and which principal factor(s) should be considered. One reason for variability in resuscitation duration and the occurrence of prolonged resuscitations may be that physicians are struggling with the numerous and highly subjective non-medical considerations elucidated in this study without a proper framework in which to address them.

PEM, EM, and non-EM physicians would perhaps benefit from this delineation of the non-medical factors often considered when terminating resuscitation in children. As discussed by Engebretse et al., simply following protocols and standards restricts imagination, reflective thinking, and critical judgment, which are all essential for objective clinical decision making. In contrast, knowledge of uncertainty lends itself to creative thinking and objective knowledge [34]. Thus, a clearer understanding of the uncertainty that exists around termination of resuscitation may help physicians to make more thoughtful and objective decisions in the unique moment when they are charged with declaring the death of a pediatric patient.

Furthermore, it has been well documented that the ED is a uniquely stressful environment and that acuity and critical events contribute toward burnout among EM physicians [35–37]. A meta-analysis by de Boer et al. discusses how work-related critical events are positively related to anxiety, depression, and even post-traumatic stress disorder (PTSD) in hospital-based health care providers [38]. Additional education about resuscitation, expanded to include the non-medical factors described here, could potentially help decrease variability and/or normalize the experiences of providers, and possibly mitigate some of the stress they experience. Likewise, these factors could provide a scaffold for self-assessment post-resuscitation and might contribute to improved mental health outcomes among physicians. Given the degree of burnout and mental health illness currently seen in the medical field [39, 40], any attempt that can be made to mitigate the contributing factors and improve the long-term health and well being of physicians would be significant.

## Limitations

As with other qualitative studies with requisite small sample sizes, the generalizability of these results is limited. PEM physicians at other hospitals may have varying perspectives due to their own clinical experiences, training, and personal beliefs and values. EM physicians who experience pediatric, adult, and geriatric arrests may have different views that were not addressed within our sample population. Furthermore, it was essential that the PI perform the interviews with participants due to the lack of an available alternative, but the familiarity between the subjects and their interviewer may have influenced participant responses in some cases.

There was a discrepancy between the number of resuscitations reported by study participants and the average number of true resuscitations that occur in the ED annually. This could be because resuscitations are memorable and impactful, leading physicians to inflate the occurrences. Another possible explanation is that participants joined another physician’s resuscitation as a co-attending and reported that resuscitation as their own, thereby increasing the number.

## Conclusion

Evidence from this study demonstrates that a variety of “non-medical” factors are considered by physicians when deciding whether to prolong or terminate resuscitation in children. Most physicians, to a certain degree, considered length of the arrest, acuity, and reversibility of the medical condition, the patient’s likely neurological outcome, parental absence at the bedside, the physician’s years of clinical experience, and the physician’s own moral and ethical perspective.

On the other hand, the data demonstrated factors less likely to be considered by the physician, which included legal and financial considerations, cultural or religious preferences of the family, whether or not the cause of arrest was non-accidental trauma, and the age of the patient.

An increased understanding of the nuanced nature of decisions regarding termination of resuscitation may help physicians in making objective clinical decisions in pediatric cardiopulmonary arrests.

## Figures and Tables

**Table 3:**
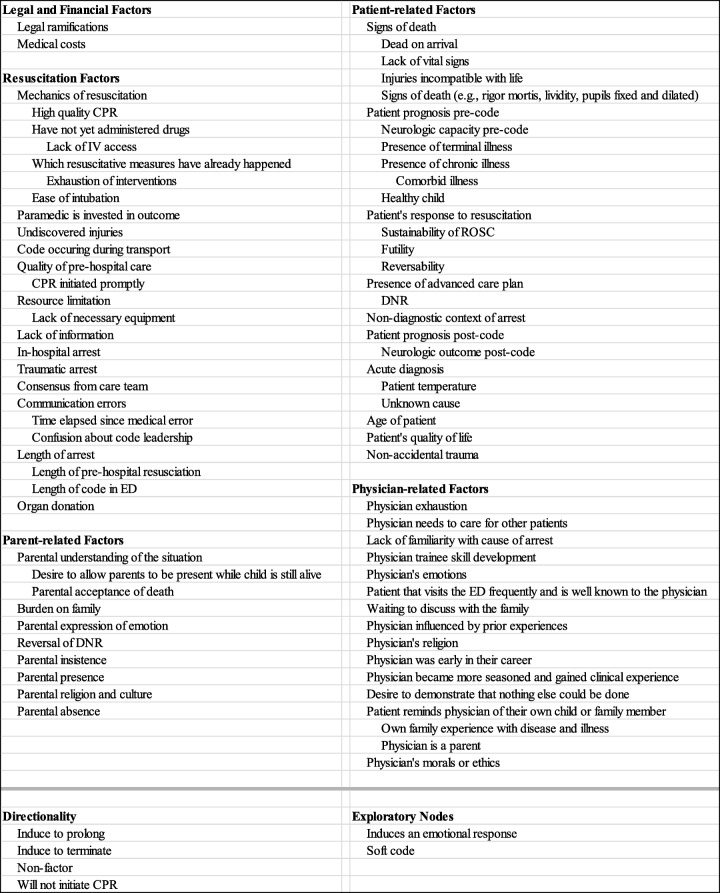
Codebook

**Table 5:**
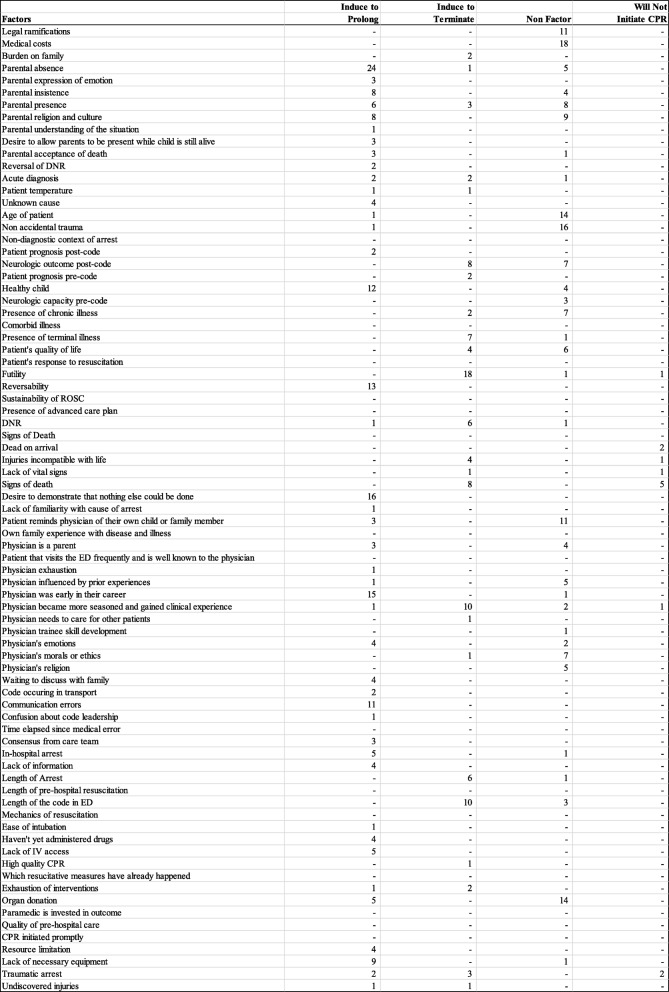
Factors and their directionality

## Appendix 1

### Interview Guide

Prompt:

The aim of this study is to identify those non-medical factors influencing a physician’s decision to terminate resuscitation in a pediatric cardiopulmonary arrest. For the purpose of this interview, we are most interested in eliciting which factors you would consider in termination of resuscitation.

Questions:

Take me back to your most memorable resuscitation (if unable to identify ask about “most recent resuscitation”).

- What factors affected your decision to terminate resuscitation?
- How typical was that? Does that come up all the time?
- Note: if participants are not addressing all of the domains, ask question(s) to illicit thought on those domains (see list below).
  - Ex: Are there ethical, moral, or spiritual factors that may contribute?
- What, if anything, would you have done differently?
- If requiring further prompting, ask about factors listed below and if these factors would affect their decision making.

Factors:

**Table:**

Patient domain	Physician domain
Patient’s age Acute condition Reversibility of condition Comorbid/chronic disease Visible tubes (trach, GTube, VPS) Total arrest time CPR initiated at the scene Dead on arrival Quality of life Neurological outcomes	Futility judgments Resuscitation/medical errors Communication among members of the resuscitation team Resource limitations Lack of family presence Presence of family Anecdotal/prior experiences Physician’s spiritual/religious belief
Parental domain	Societal domain
Advance care directives/chronic care plans Potential for organ donation Possible child abuse/homicide Parental culture practices Parental spiritual/religious beliefs	Legal ramifications Standard of care Ethical considerations Health care costs

## Appendix 3

**Table 4:** COREQ Checklist

Domain 1: Research team and reflexivity	
1. Interviewer/facilitator	RTC and a Research Coordinator conducted the interviews.
2. Credentials	RTC, TPC and ALN all are MDs. ARS has an MPH, and the Research Coordinator has a MS.
3. Occupation	All members of the research team worked at Children’s Hospital Los Angeles.
4. Gender	Both interviewers are female.
5. Experience and training	Neither interviewers had prior experience in conducting interviews, but educated themselves extensively prior to conducting the interviews. TPC has had prior experience in conducting interviews and focus groups and was able to lend expertise and guide the interviewers.
6. Relationship established	RTC was a fellow physician/trainee, and had an established relationship with the participants. Participants were familiar with the Research Coordinator, but not all had worked directly with her.
7. Participant knowledge of the interviewer	The interviewers provided a brief overview of the study prior to beginning the interview.
8. Interviewer characteristics	RTC was a fellow and had not independently terminated a resuscitation. Thus, RTC did not have opinions as to which factors are most important when deciding when to terminate a resuscitation. The Research Coordinator was a non-clinician and did not have any opinions on the subject.
Domain 2: Study design	
9. Methodological orientation and theory	Content analysis was the analytical approached used.
10. Sampling	Purposeful sampling was used to generate an information-rich group with content expertise in the subject of interest.
11. Method of approach	Physicians were invited to participate via email.
12. Sample size	17 attending physicians at the study site participated.
13. Non-participation	No subjects approached refused to participate or dropped out.
14. Setting of data collection	Data was collected at the study site, in private offices.
15. Presence of non-participants	There were no other persons present during the interviews aside from the participant and interviewers.
16. Description of sample	Demographic data is provided in Table 1.
17. Interview guide	An interview guide was created drawing from concepts from the literature and by using a modified Delphi method.
18. Repeat interviews	Repeat interviews were not a part of the protocol of this study.
19. Audio/visual recording	Audio recordings were used to collect the data.
20. Field notes	Field notes were made during the interview.
21. Duration	The interviews lasted approximately 35 minutes on average.
22. Data saturation	Data saturation was achieved.
23. Transcripts returned	Transcripts were returned to participants for comment and/or correction.
Domain 3: Analysis and findings	
24. Number of data coders	Two data coders (RTC and ARS) coded the data.
25. Description of the coding tree	A description of the coding tree is provided in the Methods and Results sections. The codebook can be found in the Appendix.
26. Derivation of themes	Themes were derived from the data.
27. Software	Nvivo version 11 Pro was used to manage the data.
28. Participant checking	Participants were provided the opportunity to provide feedback on the findings.
29. Quotations presented	See Results section.
30. Data and findings consistent	There was consistency between the data presented and the findings.
31. Clarity of major themes	Major themes were clearly presented, as illustrated by the 5 broad categories of factors outlined in the Results section.
32. Clarity of minor themes	Extensive discussion of specific cases and minor themes took place in the Results section.
